# What gets recorded, counts: dementia recording in primary care compared with a specialist database

**DOI:** 10.1093/ageing/afab164

**Published:** 2021-08-21

**Authors:** Katrina A S Davis, Christoph Mueller, Mark Ashworth, Matthew Broadbent, Amelia Jewel, Mariam Molokhia, Gayan Perera, Robert J Stewart

**Affiliations:** King's College London Institute of Psychiatry Psychology and Neuroscience, London, UK; South London and Maudsley NHS Foundation Trust, London, UK; King's College London Institute of Psychiatry Psychology and Neuroscience, London, UK; South London and Maudsley NHS Foundation Trust, London, UK; King's College London Population Health Sciences, London, UK; South London and Maudsley NHS Foundation Trust, London, UK; South London and Maudsley NHS Foundation Trust, London, UK; King's College London Population Health Sciences, London, UK; King's College London Institute of Psychiatry Psychology and Neuroscience, London, UK; South London and Maudsley NHS Foundation Trust, London, UK; King's College London Institute of Psychiatry Psychology and Neuroscience, London, UK; South London and Maudsley NHS Foundation Trust, London, UK

**Keywords:** dementia, electronic health records, primary care, cohort studies, older people

## Abstract

**Background:**

databases of electronic health records are powerful tools for dementia research, but data can be influenced by incomplete recording. We examined whether people with dementia recorded in a specialist database (from a mental health and dementia care service) differ from those recorded in primary care.

**Methods:**

a retrospective cohort study of the population covered by Lambeth DataNet (primary care electronic records) between 2007 and 2019. Documentation of dementia diagnosis in primary care coded data and linked records in a specialist database (Clinical Records Interactive Search) were compared.

**Results:**

3,859 people had dementia documented in primary care codes and 4,266 in the specialist database, with 2,886/5,239 (55%) documented in both sources. Overall, 55% were labelled as having Alzheimer’s dementia and 29% were prescribed dementia medication, but these proportions were significantly higher in those documented in both sources. The cohort identified from the specialist database were less likely to live in a care home (prevalence ratio 0.73, 95% confidence interval 0.63–0.85), have multimorbidity (0.87, 0.77–0.98) or consult frequently (0.91, 0.88–0.95) than those identified through primary care codes, although mortality did not differ (0.98, 0.91–1.06).

**Discussion:**

there is under-recording of dementia diagnoses in both primary care and specialist databases. This has implications for clinical care and for generalizability of research. Our results suggest that using a mental health database may under-represent those patients who have more frailty, reflecting differential referral to mental health services, and demonstrating how the patient pathways are an important consideration when undertaking database studies.

## Key Points

- There is evidence of under-documentation of dementia in both primary care and specialist care.
- Data from specialist providers may under-represent those with complex needs.
- Data from primary care may over-represent those who are prescribed dementia medication.
- Under-documentation may lead to less optimal clinical care and indicates possible problems in equity of care.

## Introduction

The complexity and heterogeneity of dementia means that many of the remaining research questions relating to dementia and dementia care cannot be answered by conventional means, such as randomised controlled trials. Healthcare database studies offer excellent opportunities. Many utilise electronic health records (EHR) to capture key demographic and clinical data, such as diagnostic codes, referrals and prescribing [1–3]. However, findings in healthcare databases may be influenced by where in the patient care pathway the data are collected [4, 5]. If such databases are used they may not be representative of the full population of people living with dementia, particularly in terms of clinical features. This can affect the generalisability of the findings or even the results themselves, for instance extrapolation of prevalence or absolute risk [6, 7].

In the UK, the main pathway to a dementia diagnosis, and treatment with acetylcholinesterase inhibitors for those with Alzheimer’s type dementia, is assessment in primary care followed by referral to a specialist dementia diagnostic service, often either in community mental health services or memory clinics provided by mental health services [8]. Brayne and Davis’s [6] review of sources of data for research in dementia suggests that, compared with data from primary care, data from specialist services (mental health and memory clinic providers) will tend to over-represent those who have ‘memory problems’ but are otherwise ‘relatively fit’. This study takes advantage of linked primary care EHR and specialist EHR databases to explore the degree of overlap between the cohorts of people with recorded dementia in each data source, and thus the extent of under-documentation. We explore whether the character of patients in those cohorts reflect the patient pathway such that, compared to primary care, those with dementia diagnosis in the specialist database are (i) less likely to have markers of frailty and complexity (ii) more likely to have Alzheimer’s-type dementia and be prescribed dementia medication.

## Methods

A retrospective cohort study where the cohort was patients registered with a Lambeth GP any time in the years 2007–2019, utilising linkage to a specialist database.

### Databases

Lambeth DataNet (LDN) provided data from primary care. LDN collects structured data from the EHR of all GP surgeries in the borough of Lambeth [9]. A person with a record in LDN will have had some contact with a Lambeth GP practice, which does not require residence in Lambeth.

South London and Maudsley NHS Foundation Trust (SLaM) provides specialist mental health and dementia care services for four London boroughs (Lambeth, Southwark, Lewisham and Croydon) [10]. Data from SLaM feed into a bespoke database of de-identified records through the infrastructure and oversight arrangements of the Clinical Records Interactive Search (CRIS), which can then be linked to other local and national data sources [10]. This allows the opportunity of utilising data from detailed assessments, such as those provided in memory clinics, alongside important outcomes recorded elsewhere, such as admission to general hospitals and death [11–13].

The CRIS/LDN linkage is conducted by the CRIS data-linkage service [10]. CRIS, including linkage to Lambeth DataNet, has received ethical approval as an anonymized data resource (Oxford Research Ethics Committee C, reference 18/SC/0372). This project was approved by the CRIS oversight committee. Code lists used are in Appendix 1 (ST1–6).

### Cohort

Our population was the 1.2 million people with an LDN health records between 2007 and 2019. This means we only included people who were registered with a Lambeth GP, and we included them whether or not they had a record in secondary care. We defined dementia documentation from the structured fields of the respective databases. CRIS contained ICD-10 diagnostic codes, from which we selected codes referring to dementia from the mental and behavioural disorder chapter (F00–03); LDN had Read codes and SNOMED clinical terminologies following the recent national change in preferred ontology [14]. The Read code list ascertaining dementia replicated that from the SAIL-Dementia eCohort [15] and SNOMED codes were derived from those lists using the NHS mapping file [16]. By review of the English terms attached to the Read and SNOMED Concept terms we allocated them into ‘high specificity’ (e.g. ‘Unspecified dementia’), which were sufficient on their own to indicate a diagnosis of dementia, and ‘low specificity’ (e.g. ‘Delirium superimposed on dementia’) that required supporting codes (Appendix 1, ST1).

Inclusion criteria for our main cohort were:

(1) Record for patient aged at least 18 years in LDN between 01/01/2007 and 31/05/2019.
(2a) Dementia code in CRIS (ICD-10 diagnosis fields) between 01/01/2007 and 31/05/2019.
OR
(2b) Dementia code(s) in LDN (Read or SNOMED code): either one from the ‘high specificity’ list or two different codes from the ‘low specificity’ list (ST1) with an effective date between 01/01/2007 and 31/05/2019.
AND
(3) The first recorded dementia date (recorded date for CRIS, effective date for LDN) occurred when aged 65 years or more.

### Cohort characteristics

We extracted year of birth, gender and ethnicity from LDN. When describing the denominator of people aged above 65 in LDN, we included all those with age 65 or above at the median diagnosis date of those in LDN with a diagnosis of dementia (24/05/2013). Ethnicity was assigned within LDN as 16 classes, from which we used White British unchanged as the reference class, and condensed ethnicities that may be subject to disadvantage into White non-British, Black (Black and Black British), Asian (Asian and Asian British), Mixed and Other (Chinese and Any other). LDN gives last known address at the level of Lower Super Output Areas (LSOA, a standard geographic unit with an average population of 1,700), which allowed us to calculate a neighbourhood measure of deprivation (Index of Multiple Deprivation, IMD) using publicly available data tables [17]. For sensitivity analyses, we also ascertained whether a patient lived in Lambeth and whether they had at least one consultation documented in LDN on or prior to the data of the first documentation of dementia, which we term ‘prior consultation’.

Selected health indices were extracted from LDN for dates prior to the first documentation of dementia: number of GP consultations in the previous 2 years (Appendix 1:3), smoking status (Appendix 1:4) and comorbidity score. The comorbidity score was a modified Charlson comorbidity index that used SNOMED codes for chronic conditions adapted from Read code lists developed for the CALIBER project [18] converted using the NHS mapping file [16] and summed with weights from Quan et al. [19] (excluding dementia, Appendix 1:5). Care home residence was indicated by any care home visit in consultation type in the 2 years before diagnosis.

The subtype of dementia was determined from CRIS, where possible, taking the most recent ICD-10 dementia diagnosis. Where dementia was identified in LDN only, Read/SNOMED codes that represented specific dementia subtypes were extracted from LDN (Appendix 1:2) and the most frequent subtype was allocated. Unspecified subtype was allocated where dementia was categorised as unspecified in CRIS, or no subtype codes were used in LDN. Dementia medication was defined as acetylcholinesterase inhibitors or memantine (Appendix 1:6) prescribed at least once in LDN.

### Analysis

Prevalence and patterns of missing data were explored. Descriptive statistics were calculated in MS Excel and R version 3.5.1. Confidence intervals are given around at 95% confidence (using Wilson’s method for proportions and binomial method for prevalence ratio). Proportions are given to the nearest percentage point unless <10%. Chi-squared tests were used to compare characteristics where we had specific hypotheses.

## Results

**Figure 1:**
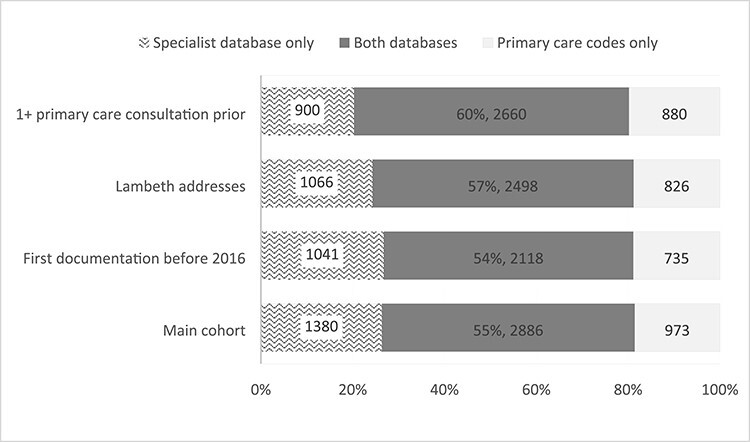
Bar chart showing the overlap of people in Lambeth DataNet identified through using linkage to a specialist mental health database and those identified through primary care codes. Results for the main cohort used in this paper compared to a number of subpopulations: First documentation before 2016 = had either specialist or primary care code between 2007 and 2015; Lambeth addresses = last known address in Lambeth; 1+ primary care consultations prior = one face-to-face or telephone encounter in primary care in the 2 years before first specialist or primary care dementia code.

Of patients with a LDN record between 2007 and 2019 aged 65 or over, 3,859 had dementia codes in primary care, with a median of two different codes from the list in Appendix 1 (interquartile range 1–7 different codes). Meanwhile 4,266 had dementia documented in the specialist care database. Combining the two sources of documentation found 5,239 unique patients with documented dementia in either source, making up 0.45% of all adult LDN patients or 5.4% of those over 65. This is our main cohort for analysis. Fifty-five percent of people identified with dementia were identified by both primary care codes and specialist database (2,886/5,239), as shown in Figure 1. 75% of those identified by primary care codes were also identified by the specialist database, and 68% of those identified by the specialist database were also identified by primary care codes. Of those identified, 84% resided in Lambeth and 85% had a prior GP consultation. Figure 1 shows the effect on overlap of restricting to these subpopulations and with a date restriction allowing for longer follow-up. Restricting the sample by residence or prior consultation modestly increased the percentage overlap in documentation from 55% to 57% (by residence, see also Appendix 2) or 60% (by prior consultation). Appendix 2 shows that both the number of cases per year and the proportion of primary care recording was highest in the years 2011–2015.

Characteristics of the main cohort are shown in Appendix 3. Three variables from LDN were found to contain missing data: ethnicity (744/5,239, 14%), smoking status (652/5,239, 12%) and LSOA/address (111/5,239, 2%). Restricting to those living in Lambeth made little difference, but prior consultation reduced the risk of missing data. Dividing the cohort into exclusive groups of those identified by both primary care codes and the specialist database (‘both’, *n* = 2,886), those identified by the specialist database only (*n* = 1,380) and those with primary care codes only (*n* = 973), levels of missing data were higher for people in the specialist only group. Tables 1 and 2 show proportions excluding missing data, while Appendix 4 shows the equivalent with missing data or restricting by prior consultation.

**Table 1:** Demographics of individuals in Lambeth DataNet in strata representing the ascertainment of dementia from two sources, specialist database and primary care codes

	Both sources	Specialist only	Primary care only
Age group in years (*n* = 5,239)	*n* = 2,880	*n* = 1,380	*n* = 973
65–74	18% (17–20)	19% (17–22)	22% (19–24)
75–84	47% (45–49)	43% (40–45)	44% (41–47)
85+	34% (33–36)	38% (35–40)	35% (32–38)
Sex (*n* = 5,239)	*n* = 2,880	*n* = 1,380	*n* = 973
Female	61% (59–63)	57% (55–60)	59% (56–62)
Male	39% (37–41)	43% (40–45)	41% (38–44)
Ethnicity (*n* = 4,495)	*n* = 2,662	*n* = 925	*n* = 908
White British	44% (42–46)	51% (48–54)+	45% (42–48)
Black	27% (26–29)	22% (19–25)–	28% (25–31)
White non-British	18% (17–20)	17% (14–19)	15% (13–18)
Asian	5.7% (4.9–6.6)	7.0% (5.6–8.9)	7.2% (5.7–9.0)
Mixed	3.0% (2.5–3.8)	2.8% (1.9–4.1)	3.0% (2.1–4.3)
Other	1.2% (0.8–1.6)	0.6% (0.3–1.4)	2.1% (1.3–3.2)
Missing	Omitted	Omitted	Omitted
Deprivation (IMD^a^) (*n* = 5,128)	*n* = 2,829	*n* = 1,345	*n* = 954
Quintile 2–5	59% (57–61)	60% (57–63)	60% (57–63)
Quintile 1 (most deprived)	41% (39–43)	40% (37–43)	40% (37–43)
Missing address	Omitted	Omitted	Omitted
Smoking (*n* = 4,589)	*n* = 2,770	*n* = 878	*n* = 941
Never	37% (35–38)	43% (40–46)+	37% (34–40)
Former^b^	48% (46–50)	37% (34–40)-	46% (43–49)
Current	15% (14–17)	20% (17–22)	18% (15–20)
Missing	Omitted	Omitted	Omitted

^a^IMD = index of multiple deprivation.

^b^Former = documented former smoking or documentation of both smoking and not smoking.

‘+’ = 95% confidence interval above values for those in ‘both’.

‘–’ = 95% confidence interval below values for those in ‘both’.

Specialist only = dementia diagnosis in specialist mental health database but no relevant primary care code; primary care only = dementia primary care code but no dementia in specialist database; both = dementia codes in both specialist database and primary care. Variable *n* due to exclusion of values that are missing. See table ST7B for inclusion of missing.

**Table 2:** Clinical characteristics of individuals in Lambeth DataNet in strata representing the ascertainment of dementia from two sources, specialist database and primary care codes

	Both sources	Specialist only	Primary care only
Primary care consultations (*n* = 5,239)^a^^,^^c^	*n* = 2,880	*n* = 1,380	*n* = 973
Above average	59% (57–60)	36% (34–39)–	50% (47–53)–
Average or less	34% (32–35)	29% (27–31)–	41% (38–44)+
None	8% (7–9)	35% (32–37)+	10% (8–12)
	*X*-squared = 584.77, df = 4, *P*-value < 0.001		
Care home residence (*n* = 5,239)^a^	*n* = 2,880	*n* = 1,380	*n* = 973
Yes	8% (7–9)	3% (3–4)-	11% (9–13)
No	92% (91–93)	97% (96–97)+	89% (87–91)
	*X*-squared = 56.377, df = 2, *P*-value < 0.001		
Comorbidity index (*n* = 5,239)^a^^,^^d^	*n* = 2,880	*n* = 1,380	*n* = 973
0	31% (30–33)	47% (45–50)+	33% (30–36)
1	22% (20–23)	20% (18–22)	19% (17–22)
2–3	34% (33–36)	24% (22–27)–	34% (31–37)
4–5	10% (9–12)	7% (6–8)–	11% (9–13)
6+	2.3% (1.8–2.9)	1.7% (1.2–2.6)	3.4% (2.4–4.7)
	*X*-squared = 121.84, df = 8, *P*-value < 0.001		
Mortality (*n* = 5,239)^b^	*n* = 2,880	*n* = 1,380	*n* = 973
No	76% (75–78)	72% (70–75)	69% (66–72)-
Yes	24% (22–25)	28% (25–30)	31% (28–34)+
	*X*-squared = 20.355, df = 2, *P*-value < 0.001		
Subtype (*n* = 4,220)	*n* = 2,644	*n* = 1,026	*n* = 550
Alzheimer’s/mixed	74% (73–76)	60% (57–63)–	52% (48–56)–
Vascular	21% (20–23)	34% (31–37)+	41% (37–45)+
Other specified	4.3% (3.6–5.2)	6.0% (4.7–7.7)	7.1% (5.2–9.5)
Unspecified	Omitted	Omitted	Omitted
	*X*-squared = 139.95, df = 4, *P*-value < 0.001		
Dementia medication (*n* = 5,239)^b^	*n* = 2,880	*n* = 1,380	*n* = 973
No	59% (57–61)	92% (91–94)+	77% (74–80)+
Yes	41% (39–43)	8% (6–9)–	23% (20–26)–
	*X*-squared = 516.79, df = 2, *P*-value < 0.0001		

Specialist only = dementia diagnosis in specialist mental health database but no relevant primary care code; primary care only = dementia primary care code but no dementia in specialist database; both = dementia codes in both specialist database and primary care. Variable *n* due to exclusion of values that are missing. See table ST7B for inclusion of missing.

^a^As documented before the first dementia documentation.

^b^In the 4 years post-first dementia documentation, or before June 2019 if earlier.

^c^Number of face to face and telephone encounters documented in Lambeth DataNet in the 2 years prior to first dementia documentation. Based on median of 23. Above average = 23+, Below average = 1–22.

^d^Modified Charlson comorbidity index from primary care-coded morbidities and weights in Quan et al. [19].

‘+’ = confidence interval above values for those in both.

‘–’ = confidence interval below values for those in both.

Table 1 shows the demographic features of the three documentation groups. The three documentation groups had similar age, sex and deprivation distribution, but ethnicity differed, with under-representation of documented Black ethnicity in those in the specialist only group. Table 2 displays the outcome of tests on the hypothesis that there was a difference between the groups on measures of frailty or complexity. A significant difference was found in all three-way comparisons (*P* < 0.001). The specialist-only group had lower Charlson comorbidity index, lower numbers of prior consultations and fewer care home consultations. The primary care only group had the highest mortality. Restricting to people who had consulted primary care in the 2 years prior to diagnosis (Appendix 4) reduced but did not abolish the differences.

Table 2 also shows that those with documentation in both databases were more commonly recorded with Alzheimer’s type dementia and less commonly documented as having ‘unspecified’ or vascular dementia than those with only one type of documentation. 29% (1,505/5,239) of patients were prescribed dementia medications in primary care, and this varied from 41% in those documented in both sources to 8% in the specialist only group. Among those prescribed dementia medication, 93% had primary care codes for dementia.

Comparing the overlapping samples of the LDN cohort that could have been generated from the specialist database (combining ‘specialist only’ and ‘both’ from Table 1, *n* = 3,859) and primary care codes (combining ‘primary care only’ and ‘both’, *n* = 4,266), Table 3 shows the specialist database sample had significantly lower proportions of White British ethnicity, lower consultation rates, lower multimorbidity and fewer in care homes—but with fairly small effect size (prevalence ratios 0.94, 0.91, 0.85, 0.73, respectively). There was no difference in mortality (prevalence ratio 0.98, 0.91–1.06). The specialist database sample are also less likely to have been prescribed dementia medication (prevalence ratio 0.85, 0.80–0.91), explored further in Appendix 5, which shows the largest discrepancy in being prescribed medication was in those with Alzheimer’s type dementia.

**Table 3:** Comparison of characteristics of individuals in Lambeth DataNet in two overlapping cohorts: dementia documented in specialist database; and dementia codes in LDN/primary care

	Prevalence in patients identified by specialist database (+/− primary care codes)		Prevalence in patients identified by primary care codes (+/− specialty database)		Prevalence ratio—specialist sample: primary care sample
Age group (years): 85+	1,516/4,266	35.5%	1,333/3,859	34.5%	1.03 (0.97–1.09)
Sex: Female	2,557/4,266	59.9%	2,340/3,859	60.6%	0.99 (0.95–1.02)
Ethnicity: White British	1,650/4,266	38.7%	1,587/3,859	41.1%	0.94 (0.89–0.99)
Relative frailty					
Comorbidity high^a^	484/4,266	11.3%	503/3,859	13.0%	0.87 (0.77–0.98)
Prior consultation high^b^	2,190/4,266	51.3%	2172/3,859	56.3%	0.91 (0.88–0.95)
Care home^c^	270/4,266	6.3%	334/3,859	8.7%	0.73 (0.63–0.85)
Death during follow-up^d^	1,073/4,266	25.2%	990/3,859	25.7%	0.98 (0.91–1.06)
Subtype					
Alzheimer’s/mixed^e^	2,577/4,266	60.4%	2,249/3,859	58.3%	1.04 (1.00–1.07)
Dementia medication^f^	1,076/3,564	30.2%	1,176/3,324	35.4%	0.85 (0.80–0.91)

^a^Scored 4 or more on the modified Charlson comorbidity index (ST5).

^b^In 2 years prior to first dementia diagnosis had face to face or telephone consultation with primary care at or above median number of 23.

^c^At least one consultation marked as occurring in a care home at any time prior to first dementia diagnosis.

^d^Death recorded within 4 years of first dementia diagnosis or 31/05/2019 if earlier.

^e^86% of those in CRIS cohort and 82% of those in GP cohort had subtype recorded. See Appendix 4 for more details.

^f^Prescribed AChEI or memantine by GP in the 4 years following first dementia documentation or before June 2019 if earlier.

## Discussion

We investigated the likely generalisability of findings made from databases of routinely recorded healthcare data by assessing patient characteristics associated with cohorts derived from two methods of ascertaining dementia cases in a defined population: structured diagnosis in a specialist mental health dementia service and coded documentation in primary care. We identified 5,239 patients with eligible dementia documentation, 55% of whom were documented in both data sources, 26% only in specialist care and 19% only in primary care. Those with dementia documented in the specialty database were less likely to live in a care home, consult the GP less frequently and have fewer comorbidities than those with dementia documented in the primary care codes. It therefore seems likely that the specialist database under-reflects frail and complex patients. Perhaps surprisingly, those in the specialist database were not more likely to have Alzheimer’s dementia and they were less likely to be prescribed dementia medication.

Both NICE guidelines and the primary care services contract emphasise the need for full memory clinic assessment in most cases when dementia is suspected, and that the clinic will assess for suitability for medication [8, 20], which led to our hypothesis that we would see over-representation of those prescribed dementia medication in the specialist database. However, 93% of those prescribed dementia medication had primary care coding, compared with 66% of those not prescribed dementia medication. This may be due to reverse causation—those patients prescribed dementia medications by their GP subsequently get coded with dementia. Those without diagnosis in the specialist database, some of whom were prescribed dementia medications, might reflect diagnosis in other places such as clinics for the care of older people (not included in our data-source), which may be deemed more appropriate if patients had a mixture of physical and cognitive difficulties.

Of the people with dementia documented in the specialist database, 32% did not have this formally documented in primary care; this despite pressure on GPs to recognise possible dementia, refer and document diagnosis [20]. Our work is consistent with others in that primary care documentation increased in 2011–2015 when specific funding was available for dementia case finding [21–23], but that gaps in documentation remain. For example, comparing general hospital statistics with primary care codes has shown proportions of cases with a dementia diagnosis on their hospital data that did not have this recorded in primary care was 44% in an English sample [24] and 39% in Wales [15]. Severity is thought to be a predictor of documentation in primary care [13, 25], to which we can add prescription of dementia medication. Our results suggest that White British people are more likely to have primary care codes than those of Black ethnicity—although our study was not looking at this, and so the finding should be regarded as tentative. Some under-ascertainment may occur when people move in and out of areas (for example to enter a care home), as GP practices in the UK each have their own electronic records that may not move with the patient or integrate with other IT systems. Under-documentation is a barrier to good clinical care [12, 26]. Initiatives are consequently being developed to integrate care records to ensure clinicians have the information they need wherever the patient presents [27, 28].

Under-documentation will have obvious repercussions on estimating the prevalence of diagnosed dementia, but a lack of sensitivity has wider consequences for research [29]. Unless a source of dementia diagnosis is near-complete, identification of people with dementia using this documentation will reflect patient and system factors that influenced the documentation, with risk of misclassification in the study. Our findings indicate that when patients with dementia are selected using single agency data the cohort may not be fully representative in both demographics and clinical characteristics. Conversely, these findings may indicate that the patient pathways themselves are not delivering equity of access.

### Strengths and limitations

To our knowledge, this is the first study to compare dementia recorded in primary and specialist care in the UK. While the exact findings may not be generalisable elsewhere (especially due to the populations served in this catchment [10]), we expect the observations about under-documentation will be widely applicable. We used previously applied code lists to maximise the applicability of findings; however, limiting to coded data may have under-ascertained dementia documented as free-text. For comorbidities, we took lack of documentation to mean absence of condition, but they will be subject to the same under-documentation biases as we describe for dementia. For prescribing, we are assuming that specialist services always asked primary care to prescribe dementia medications (as was the policy), but there may have been patients who received it directly. Our inclusion criteria included people who were registered with a GP practice in Lambeth for only part of the date window, which may have accounted for another portion of under-ascertainment. Including more data sources to our search (such as from general hospitals in the area) may have increased the number of individuals we identified, and would be likely to show more under-documentation.

Any documentation of dementia that met our criteria was taken to represent a true positive case of dementia, but the code lists and our algorithm have not been externally validated against a clinical assessment. Given the relatively high prevalence of dementia in older adults and the known problem of under-documentation [5] we assume that false negatives are more likely than false positives as a cause for lack of overlap, but it is likely that there are also cases of mistakes in documentation. We are also conscious that the documentation gap we have demonstrated is related, but separate to, the diagnosis gap. To fully understand the under-documentation for people with dementia, we would need to include a cohort screened for dementia to identify those without diagnosis.

### Conclusions and implications

Documentation in EHR is important for clinical care and secondary use for database research studies. We found that two EHR databases for the same population sample found broadly equal numbers of people documented as living with dementia with substantial, but incomplete, overlap in the people identified. This incomplete documentation may suggest some inequality of access, which deserves further investigation. Researchers and clinicians using healthcare databases should be aware that where they cover only some of the real-life patient pathways, they may miss a proportion of people with dementia, and take this into account when choosing databases and interpreting the results. Opportunities for data linkage drawing from multiple databases will improve the generalisability of findings.

## Supplementary Material

aa-21-0495-File002_afab164

## References

[1] Joshi I, Morley J, NHSX; Artificial Intelligence: How to Get it Right, Putting Policy into Practice for Safe Data-Driven Innovation in Health and Care. London, United Kingdom: NHSX. https://www.nhsx.nhs.uk/media/documents/NHSX_AI_report.pdf, 2019.

[2] Bauermeister S, Orton C, Thompson S et al. The Dementias Platform UK (DPUK) Data Portal. Eur J Epidemiol 2020; 35: 601–11. 32328990 10.1007/s10654-020-00633-4 PMC7320955

[3] Davis KA, Farooq S, Hayes JF et al. Pharmacoepidemiology research: delivering evidence about drug safety and effectiveness in mental health. Lancet Psychiatry 2019; 7: 363–70. 31780306 10.1016/S2215-0366(19)30298-6

[4] Goldberg D, Huxley P. Mental Illness in the Community: the Pathway to Psychiatric Care. Psychology Press, 2001.

[5] Wilkinson T, Ly A, Schnier C et al. Identifying dementia cases with routinely collected health data: a systematic review. Alzheimers Dement 2018; 14: 1038–51. 29621480 10.1016/j.jalz.2018.02.016 PMC6105076

[6] Brayne C, Davis D. Making Alzheimer's and dementia research fit for populations. Lancet 2012; 380: 1441–3. 23084456 10.1016/S0140-6736(12)61803-0 PMC3974586

[7] Sørensen HT, Sabroe S, Olsen J. A framework for evaluation of secondary data sources for epidemiological research. Int J Epidemiol 1996; 25: 435–42. 9119571 10.1093/ije/25.2.435

[8] National Institute for Health and Care Excellence (NICE) . Dementia: assessment, management and support for people living with dementia and their carers. NICE Guideline number 97; 2018. https://www.nice.org.uk/guidance/ng97/evidence/full-guideline-pdf-48526. 30011160

[9] Lambeth Clinical Commissioning Group . CCG uses patient data to develop highly targeted initiatives aimed at many different conditions and treatments, resulting in improved understanding of conditions and reducing variations in care. In: Solutionss HSJ, ed. Value in Healthcare Awards. London: Wilmington, 2017.

[10] Perera G, Broadbent M, Callard F et al. Cohort profile of the South London and Maudsley NHS Foundation Trust Biomedical Research Centre (SLaM BRC) case register: current status and recent enhancement of an electronic mental health record-derived data resource. BMJ Open 2016; 6: e008721. 10.1136/bmjopen-2015-008721 PMC4785292 26932138

[11] Mueller C, Molokhia M, Perera G et al. Polypharmacy in people with dementia: associations with adverse health outcomes. Exp Gerontol 2018; 106: 240–5. 29452289 10.1016/j.exger.2018.02.011

[12] Sommerlad A, Perera G, Mueller C et al. Hospitalisation of people with dementia: evidence from English electronic health records from 2008 to 2016. Eur J Epidemiol 2019; 34: 567–77. 30649705 10.1007/s10654-019-00481-x PMC6497615

[13] Gungabissoon U, Perera G, Galwey NW et al. The association between dementia severity and hospitalisation profile in a newly assessed clinical cohort: the South London and Maudsley case register. BMJ Open 2020; 10: e035779. 10.1136/bmjopen-2019-035779. PMC7200045 32284392

[14] Wardle M, Spencer A. Implementation of SNOMED CT in an online clinical database. Future Healthc J 2017; 4: 126–30. 10.7861/futurehosp.4-2-126 PMC6502621 31098449

[15] Schnier C, Wilkinson T, Orton C et al. The secure anonymised information linkage databank dementia e-cohort (SAIL-DeC). Int J Population Data Sci 2019; 4. 10.23889/ijpds.v5i1.1121. PMC7473277 32935048

[16] TRUD . READ Codes. London: NHS Digital. 2019. https://isd.digital.nhs.uk/trud3/user/guest/group/0/pack/9/subpack/9/releases (2020, date last accessed).

[17] Ministry of Housing Communities and Local Government. London: Office of National Statistics. English indices of deprivation 2015. https://www.gov.uk/government/statistics/english-indices-of-deprivation-2015 (22 July 2021, date last accessed).

[18] University College London . CALIBER. https://www.ucl.ac.uk/health-informatics/caliber (2020, date last accessed).

[19] Quan H, Li B, Couris CM et al. Updating and validating the charlson comorbidity index and score for risk adjustment in hospital discharge abstracts using data from 6 Countries. Am J Epidemiol 2011; 173: 676–82. 21330339 10.1093/aje/kwq433

[20] Primary Care Strategy and NHS Contracts Group . Dementia (DEM). In: BMA, NHS Englands, ed. 2019/20 General Medical Services (GMS) contract Quality and Outcomes Framework (QOF). #NHSLongTermPlan, 2019; 59–60.

[21] Liu D, Green E, Kasteridis P et al. Incentive schemes to increase dementia diagnoses in primary care in England: a retrospective cohort study of unintended consequences. Brit J Gen Pract 2019; 69: e154–63. 30803980 10.3399/bjgp19X701513 PMC6400615

[22] Brayne C . Interpretation of dementia diagnosis and treatment trends in the UK over time. Lancet Public Health 2017; 2: e128–9. 29253384 10.1016/S2468-2667(17)30030-0

[23] Donegan K, Fox N, Black N et al. Trends in diagnosis and treatment for people with dementia in the UK from 2005 to 2015: a longitudinal retrospective cohort study. Lancet Public Health 2017; 2: e149–56. 29253388 10.1016/S2468-2667(17)30031-2

[24] Pujades-Rodriguez M, Assi V, Gonzalez-Izquierdo A et al. The diagnosis, burden and prognosis of dementia: a record-linkage cohort study in England. PloS one 2018; 13: e0199026. 29944675 10.1371/journal.pone.0199026 PMC6019102

[25] Zhu CW, Ornstein KA, Cosentino S et al. Misidentification of dementia in medicare claims and related costs. J Am Geriatr Soc 2019; 67: 269–76. 30315744 10.1111/jgs.15638 PMC6429550

[26] Burn A-M, Fleming J, Brayne C et al. Dementia case-finding in hospitals: a qualitative study exploring the views of healthcare professionals in English primary care and secondary care. BMJ Open 2018; 8. 10.1136/bmjopen-2017-020521. PMC5875605 29550782

[27] Singh I . So, what is a local health and care record anyway? In: Transformation Blog, ed. NHS Digital, 2019. https://digital.nhs.uk/blog/transformation-blog/2019/so-what-is-a-local-health-and-care-record-anyway.

[28] North London Partners ; OneLondon. https://www.northlondonpartners.org.uk/ourplan/Areas-of-work/Digital/one-london.htm (22 July 2021 date last accessed).

[29] McGuinness LA, Warren-Gash C, Moorhouse LR et al. The validity of dementia diagnoses in routinely collected electronic health records in the United Kingdom: a systematic review. Pharmacoepidemiol Drug Saf 2019; 28: 244–55. 30667114 10.1002/pds.4669 PMC6519035

