# Supplementary material for: What gets recorded, counts: dementia recording in primary care compared with a specialist database
Source: Age Ageing. 2021 Aug 21;50(6):2206–13. doi: 10.1093/ageing/afab164 (PMC8581382; doi:10.1093/ageing/afab164)
Supplement: aa-21-0495-File002_afab164 [file aa-21-0495-file002_afab164.docx]

What gets recorded, counts: Dementia recording in primary care compared with a specialist database

**SUPPLEMENTARY DATA**

Contents

[Appendix 1: Lists used in study 2](#_Toc72242851)

[Appendix 2: [table] Proportion of people with dementia identified by two sources looking over time and by Lambeth residence 15](#_Toc72242852)

[Appendix 3: [table] Characteristics and missing data for main cohort from Lambeth DataNet, compared against those also with Lambeth residence. 16](#_Toc72242853)

[Appendix 4: [table] Characteristics of patients with dementia documented in either specialist database or primary care database or both 18](#_Toc72242854)

[Appendix 5: [table&figure] Prescription of dementia medication in primary care by source of dementia documentation and subtype of dementia 22](#_Toc72242855)

# Appendix 1: Lists used in study

**Code lists used in: What gets recorded, counts: Dementia recording in primary care compared with a specialist database.**

Note: 5-byte Read codes used as standard. SNOMED codes allocated using NHS linking file, and should be regarded as approximation of the original Read code.

An excel file with complete code lists is also available DOI: [10.13140/RG.2.2.14629.42729](http://dx.doi.org/10.13140/RG.2.2.14629.42729)

**Contents:**

A1 Supplementary table 1: Primary care dementia documentation

A1 Supplementary table 2: Primary care documentation of dementia subtypes

A1 Supplementary table 3: Consultation types in Lambeth DataNet

A1 Supplementary table 4: Smoking status documentation

A1 Supplementary table 5: Charlson comorbidity index overview

A1 Supplementary table 6: Dementia medication

A1 Supplementary table 1: **Primary care dementia documentation**

| Source: adapted from the SAIL Dementia eCohort (Schnier C, Wilkinson T, Orton C, North L, Rochford R, Sudlow C. The Secure Anonymised Information Linkage databank Dementia e-cohort (SAIL-DeC). International Journal of Population Data Science. 2019;4(3).) | | | |
| --- | --- | --- | --- |
| https://static-content.springer.com/esm/art%3A10.1007%2Fs10654-019-00499-1/MediaObjects/10654_2019_499_MOESM1_ESM.pdf | | | |
|  |  |  |  |
| Description | Read code | SNOMED Concept | "High" or "Low" specificity code |
| [X] Dementia in Alzheimer's disease | Eu00. | 142811000119104 | High |
| [X]Dementia in Alzheimer's disease with early onset | Eu000 | 416780008 | High |
| [X]Dementia in Alzheimer's disease with late onset | Eu001 | 416975007 | High |
| [X]Dementia in Alzheimer's disease, atypical or mixed type | Eu002 | 419261000000107 | High |
| [X]Dementia in Alzheimer's disease, unspecified | Eu00z | 26929004 | High |
| Alzheimer’s disease | F110. | 26929004 | High |
| Alzheimer’s disease with early onset | F1100 | 416780008 | Low |
| Alzheimer’s disease with late onset | F1101 | 416975007 | High |
| Senile degeneration of brain | F112. | 45864009 | Low |
| [X] Other Alzheimer's disease | Fyu30 | 26929004 | High |
| Multi-infarct dementia | E004. | 429998004 | High |
| Uncomplicated arteriosclerotic dementia | E0040 | 191463004 | High |
| Arteriosclerotic dementia with delirium | E0041 | 191464005 | High |
| Arteriosclerotic dementia with paranoia | E0042 | 191465006 | High |
| Arteriosclerotic dementia with depression | E0043 | 191466007 | High |
| Arteriosclerotic dementia NOS | E004z | 56267009 | High |
| [X]Vascular dementia | Eu01. | 429998004 | High |
| [X]Vascular dementia of acute onset | Eu010 | 230285003 | High |
| [X]Multi-infarct dementia | Eu011 | 56267009 | High |
| [X]Other vascular dementia | Eu01y | 429998004 | High |
| [X]Vascular dementia, unspecified | Eu01z | 429998004 | High |
| Cerebral degeneration due to cerebrovascular disease | F11x2 | 192813004 | Low |
| Binswanger's disease | F21y2 | 90099008 | High |
| [X] Lewy body dementia | Eu025 | 312991009 | High |
| Lewy body disease | F116. | 312991009 | High |
| [X] Dementia in Picks disease | Eu020 | 21921000119103 | High |
| Pick's disease | F111. | 13092008 | High |
| Frontotemporal degeneration | F118. | 230273006 | Low |
| Jakob-Creutzfeldt disease | A411. | 792004 | Low |
| Sporadic Creutzfeldt-Jakob disease | A4110 | 713060000 | Low |
| Alcoholic dementia, NOS | E012. | 281004 | Low |
| Dementia in conditions EC | E041. | 191519005 | High |
| [X] Dementia in other diseases classified elsewhere | Eu02. | 191519005 | High |
| [X] Dementia in Creutzfeldt-Jacob disease | Eu021 | 429458009 | High |
| [X] Dementia in Huntington’s disease | Eu022 | 442344002 | High |
| [X] Dementia in Parkinson’s disease | Eu023 | 425390006 | High |
| [X] Dementia in HIV disease | Eu024 | 421529006 | High |
| [X]Dementia in other specified diseases classified elsewhere | Eu02y | 191519005 | High |
| [X]Mental and behavioural disorders due to use of alcohol: amnesic syndrome | Eu106 | 73097000 | Low |
| [X]Mental and behavioural disorders due to use of alcohol: residual and late-onset psychotic disorder | Eu107 | 281004 | Low |
| Cerebral degeneration due to Jacob-Creutzfeldt disease | F11x7 | 192818008 | Low |
| Cerebral degeneration due to Parkinson’s disease | F11x9 | 341551000000108 | Low |
| Corticobasal degeneration | F11y2 | 18842008 | Low |
| H/O: dementia | 1461 | 161465002 | High |
| Assessment of psychotic and behavioural symptoms of dementia | 38C13 | 700464008 | Low |
| GDS level 4 - moderate cognitive decline | 3AE3. | 407632003 | High |
| GDS level 5 - moderately severe cognitive decline | 3AE4. | 407633008 | High |
| GDS level 6 - severe cognitive decline | 3AE5. | 407634002 | High |
| GDS level 7 - very severe cognitive decline | 3AE6. | 407635001 | High |
| Dementia monitoring | 66h.. | 248711000000102 | High |
| Dementia annual review | 6AB.. | 249181000000100 | Low |
| Dementia medication review | 8BM02 | 938551000000108 | Low |
| Shared care – prescribing drug for dementia | 8BM50 | 719787003 | High |
| Shared care – prescribing drug for dementia declined | 8BM60 | 720022007 | High |
| Antipsyc drug therapy dementia | 8BPa. | 700214004 | High |
| Dementia advance care plan | 8CMe0 | 959361000000105 | Low |
| Review of dementia advance care plan | 8CMG2 | 713580008 | Low |
| Dementia care plan | 8CMZ. | 736371006 | Low |
| Dementia care plan agreed | 8CMZ0 | 956841000000106 | Low |
| Dementia care plan reviewed | 8CMZ1 | 956861000000107 | Low |
| Dementia care plan declined | 8CMZ2 | 956881000000103 | Low |
| Dementia care plan review declined | 8CMZ3 | 956901000000100 | Low |
| Dementia advance care plan agreed | 8CSA. | 1095121000000100 | Low |
| Referral to dementia care advisor | 8Hla. | 798381000000109 | Low |
| Dementia adv care plan declnd | 8IAe0 | 956881000000103 | Low |
| Dementia advance care plan review declined | 8IAe2 | 959461000000102 | Low |
| Exception reporting: dementia quality indicators | 9hD.. | 715881000000108 | Low |
| Excepted from dementia quality indicators: patient unsuitable | 9hD0. | 716341000000104 | Low |
| Excepted from dementia quality indicators: informed dissent | 9hD1. | 716131000000105 | Low |
| Dementia monitoring administration | 9Ou.. | 713821000000106 | Low |
| Dementia monitoring first letter | 9Ou1. | 715821000000107 | Low |
| Dementia monitoring second letter | 9Ou2. | 717471000000101 | Low |
| Dementia monitoring third letter | 9Ou3. | 716671000000102 | Low |
| Dementia monitoring verbal invite | 9Ou4. | 716221000000104 | Low |
| Dementia monitoring telephone invite | 9Ou5. | 716991000000108 | Low |
| Senile and presenile organic psychotic condition | E00.. | 15662003 | Low |
| Uncomplicated senile dementia | E000. | 191449005 | High |
| Pre-senile dementia | E001. | 12348006 | Low |
| Uncomplicated pre-senile dementia | E0010 | 191451009 | Low |
| Pre-senile dementia with delirium | E0011 | 191452002 | Low |
| Pre-senile dementia with paranoia | E0012 | 191454001 | Low |
| Pre-senile dementia with depression | E0013 | 191455000 | Low |
| Pre-senile dementia NOS | E001z | 12348006 | Low |
| Senile dementia with depressive or paranoid features | E002. | 191457008 | High |
| Senile dementia with paranoia | E0020 | 191458003 | High |
| Senile dementia with depression | E0021 | 191459006 | High |
| Senile dementia with depressive or paranoid features NOS | E002z | 191457008 | High |
| Senile dementia with delirium | E003. | 191461002 | Low |
| Drug induced dementia | E02y1 | 191493005 | Low |
| [X]Sub-cortical vascular dementia | Eu012 | 230286002 | High |
| [X]Mixed cortical and sub-cortical vascular dementia | Eu013 | 230287006 | High |
| [X] Unspecified dementia | Eu02z | 22381000119105 | High |
| [X] Delirium superimposed on dementia | Eu041 | 2776000 | Low |

A1 Supplementary table 2: **Primary care documentation of dementia subtypes (all occur in above dementia documentation). Key: AD = Alzheimer's-type dementia, VD = Vascular dementia, O = Dementia of other type**

| Source: https://static-content.springer.com/esm/art%3A10.1007%2Fs10654-019-00499-1/MediaObjects/10654_2019_499_MOESM1_ESM.pdf | | | |
| --- | --- | --- | --- |
|  |  |  |  |
| **Description** | **Read code** | **SNOMED Concept** | **Subtype** |
| [X] Dementia in Alzheimer's disease | Eu00. | 142811000119104 | AD |
| [X]Dementia in Alzheimer's disease with early onset | Eu000 | 416780008 | AD |
| [X]Dementia in Alzheimer's disease with late onset | Eu001 | 416975007 | AD |
| [X]Dementia in Alzheimer's disease, atypical or mixed type | Eu002 | 419261000000107 | AD |
| [X]Dementia in Alzheimer's disease, unspecified | Eu00z | 26929004 | AD |
| Alzheimer’s disease | F110. | 26929004 | AD |
| Alzheimer’s disease with early onset | F1100 | 416780008 | AD |
| Alzheimer’s disease with late onset | F1101 | 416975007 | AD |
| Senile degeneration of brain | F112. | 45864009 | AD |
| [X] Other Alzheimer's disease | Fyu30 | 26929004 | AD |
| Multi-infarct dementia | E004. | 429998004 | VD |
| Uncomplicated arteriosclerotic dementia | E0040 | 191463004 | VD |
| Arteriosclerotic dementia with delirium | E0041 | 191464005 | VD |
| Arteriosclerotic dementia with paranoia | E0042 | 191465006 | VD |
| Arteriosclerotic dementia with depression | E0043 | 191466007 | VD |
| Arteriosclerotic dementia NOS | E004z | 56267009 | VD |
| [X]Vascular dementia | Eu01. | 429998004 | VD |
| [X]Vascular dementia of acute onset | Eu010 | 230285003 | VD |
| [X]Multi-infarct dementia | Eu011 | 56267009 | VD |
| [X]Other vascular dementia | Eu01y | 429998004 | VD |
| [X]Vascular dementia, unspecified | Eu01z | 429998004 | VD |
| Cerebral degeneration due to cerebrovascular disease | F11x2 | 192813004 | VD |
| Binswanger's disease | F21y2 | 90099008 | VD |
| [X] Lewy body dementia | Eu025 | 312991009 | O |
| Lewy body disease | F116. | 312991009 | O |
| [X] Dementia in Picks disease | Eu020 | 21921000119103 | O |
| Pick's disease | F111. | 13092008 | O |
| Frontotemporal degeneration | F118. | 230273006 | O |
| Alcoholic dementia, NOS | E012. | 281004 | O |
| [X]Mental and behavioural disorders due to use of alcohol: amnesic syndrome | Eu106 | 73097000 | O |
| [X]Mental and behavioural disorders due to use of alcohol: residual and late-onset psychotic disorder | Eu107 | 281004 | O |

A1 Supplementary table 3: **Consultation types in Lambeth DataNet**

| Source: Generated internally |  |  |
| --- | --- | --- |
| **ConsultationTypeTerm** | **N_total in LDN** | **included** |
| GP Surgery | 30047521 | Y |
| Externally entered note | 11897955 | N |
| Administration note | 8104352 | N |
| Scanned document | 7297117 | N |
| Patient encounter data NOS | 5699995 | N |
| Telephone consultation | 3227510 | Y |
| Laboratory result | 2357993 | N |
| NULL | 1958780 | N |
| Face to face consultation | 613991 | Y |
| Mail from patient | 402619 | N |
| Other note | 389818 | N |
| Hospital outpatient report | 341925 | N |
| Home visit note | 259265 | Y |
| Non-consultation data | 248453 | N |
| Telephone call to a patient | 243062 | Y |
| Non-consultation medication data | 212227 | N |
| Telephone encounter | 209876 | Y |
| Did not attend | 186524 | N |
| Inbound document | 156727 | N |
| Walk-in clinic | 143437 | Y |
| Telephone triage encounter | 138996 | Y |
| Urgent consultation | 112410 | Y |
| Emergency consultation | 112370 | Y |
| Night visit note | 98111 | Y |
| Indirect encounter | 90961 | N |
| Repeat prescription | 90489 | N |
| SMS text message sent to patient | 65309 | N |
| OOH report | 47896 | N |
| Discussion with colleague | 37663 | N |
| Extended hours consultation | 23731 | Y |
| Telephone call to relative/carer | 20565 | N |
| Mail to patient | 20152 | N |
| E-mail consultation | 19515 | N |
| Hospital inpatient report | 18553 | N |
| Seen in baby clinic | 11996 | N |
| Telephone call from relative/carer | 8936 | N |
| **Residential home visit note** | **8544** | **Y** |
| Online communication | 8102 | N |
| Other consultation medium used | 7328 | Y |
| E-mail received from patient | 6086 | N |
| Group consultation | 4643 | N |
| E-mail sent to patient | 4326 | N |
| Seen in private clinic | 3208 | N |
| Case conference | 2839 | N |
| Emergency appointment | 2763 | Y |
| Seen in asthma clinic | 1589 | Y |
| School visit note | 1583 | N |
| Multidisciplinary team meeting with patient | 1200 | Y |
| Discussion with other professional | 1036 | N |
| Face to face consultation with relative/carer | 805 | N |
| Same day appointment | 699 | Y |
| Children's home visit note | 688 | N |
| Enterprise consultation | 533 | N |
| Hospital inpatient note | 516 | N |
| Consultation via telemedicine web camera | 514 | N |
| Consultation via multimedia | 292 | N |
| Routine consultation | 269 | Y |
| Seen in GP unit | 251 | Y |
| Radiology result | 241 | N |
| Pharmacy consultation | 175 | N |
| Nurse telephone triage | 134 | N |
| Multidisciplinary team meeting without patient | 116 | N |
| First attendance face to face | 100 | Y |
| Follow up attendance face to face | 95 | Y |
| Genito-urinary medicine | 87 | N |
| Medication requested | 34 | N |
| Inbound referral | 30 | N |
| E-mail received from carer | 27 | N |
| Outbound referral | 21 | N |

A1 Supplementary table 4: **Smoking status documentation**

**Key: 1 = current smoker strong code, 2 = current smoker weak code, 3 = Ever smoker, 4 = Not current, 5 = Never smoked**

Algorithm used: *Sum up number in each category. If category (1) has the most and there are no (5), then* ***current smoker****. If category (5) has the most and there are no (1) then* ***never smoker****. All other are* ***mixed/former*** *smoker*.

| Collated by Katrina Davis (katrina.davis@kcl.ac.uk) and Catherine Polling | | | |
| --- | --- | --- | --- |
| **Description** | **Read code** | **SNOMEDConcept**  **NA = no match** | **Smoking status** |
| Smoking cessation advice | 8CAL. | 225323000 | 1 |
| Cigarette smoker | 137P. | 65568007 | 1 |
| Current smoker | 137R. | 77176002 | 1 |
| Moderate smoker - 10-19 cigs/d | 1374. | 160604004 | 1 |
| Light smoker - 1-9 cigs/day | 1373. | 160603005 | 1 |
| Heavy smoker - 20-39 cigs/day | 1375. | 160605003 | 1 |
| Trivial smoker - < 1 cig/day | 1372. | 428041000124106 | 1 |
| Rolls own cigarettes | 137M. | 160619003 | 1 |
| Smoking cessation advice declined | 8IAj. | 527151000000107 | 1 |
| Referral to smoking cessation advisor | 8H7i. | 395700008 | 1 |
| Cigar smoker | 137J. | 59978006 | 1 |
| Pipe smoker | 137H. | 82302008 | 1 |
| Not interested in stopping smoking | 137d. | 394873005 | 1 |
| Lifestyle advice regarding smoking | 67H1. | 225323000 | 1 |
| Smoking cessation drug therapy declined | 8IEM. | 822591000000108 | 1 |
| Thinking about stopping smoking | 137c. | 394871007 | 1 |
| Smoking cessation programme declined | 8IEK. | 1087441000000100 | 1 |
| Very heavy smoker - 40+cigs/d | 1376. | 160606002 | 1 |
| Failed attempt to stop smoking | 137m. | 446172000 | 1 |
| Keeps trying to stop smoking | 137C. | 160612007 | 1 |
| Stop smoking service opportunity signposted | 8CdB. | 783011000000105 | 1 |
| Tobacco dependence | E251. | 89765005 | 1 |
| Smoking reduced | 137V. | 134406006 | 1 |
| Current smoker annual review - enhanced services administration | 9ko.. | 505651000000103 | 1 |
| Smoking restarted | 137e. | 308438006 | 1 |
| [V]Tobacco use | ZV4K0 | 110483000 | 1 |
| Smoking cessation advice provided by community pharmacist | 8CAg. | 200221000000105 | 1 |
| Minutes from waking to first tobacco consumption | 137h. | 413173009 | 1 |
| Reason for restarting smoking | 137f. | 401159003 | 1 |
| [X]Mental and behavioural disorders due to use of tobacco: harmful use | Eu171 | 470041000000100 | 1 |
| [V]Tobacco abuse counselling | ZV6D8 | 711028002 | 1 |
| [X]Mental and behavioural disorders due to use of tobacco: acute intoxication | Eu170 | 466951000000105 | 1 |
| Tobacco dependence, continuous | E2511 | 191887008 | 1 |
| [X]Mental and behavioural disorder due to use of tobacco | Eu17. | 30310000 | 1 |
| Varenicline smoking cessation therapy declined | 8IEM0 | 966971000000103 | 1 |
| Tobacco consumption | 137.. | 266918002 | 2 |
| Tobacco consumption NOS | 137Z. | 266918002 | 2 |
| Stop smoking monitor 1st lettr | 9OO4. | 185792005 | 2 |
| Cigarette consumption | 137X. | 230056004 | 2 |
| Smoking started | 137Q. | 266929003 | 2 |
| Pipe tobacco consumption | 137a. | 230058003 | 2 |
| Stop smoking monitor 2nd lettr | 9OO5. | 185793000 | 2 |
| Stop smoking monitor verb.inv. | 9OO7. | 185795007 | 2 |
| Cigar consumption | 137Y. | 230057008 | 2 |
| Attends stop smoking monitor. | 9OO1. | 185789006 | 2 |
| Refuses stop smoking monitor | 9OO2. | 765001003 | 2 |
| Smokers’ cough | H3101 | 46802002 | 2 |
| Stop smoking monitor 3rd lettr | 9OO6. | 185794006 | 2 |
| Stop smoking monitor phone inv | 9OO8. | 185796008 | 2 |
| Stop smoking monitor.chck done | 9OOA. | 185799001 | 2 |
| Waterpipe tobacco consumption | 137o. | 836001000000109 | 2 |
| Stop smoking invitation first short message service text message | 9OOB0 | 783401000000101 | 2 |
| Chronic obstructive pulmonary disease structured smoking assessment declined - enhanced services administration | 9kf2. | 375911000000102 | 2 |
| Stop smoking invitation third short message service text message | 9OOB2 | 783481000000106 | 2 |
| Fagerstrom test for nicotine dependence | 38DH. | 1084701000000100 | 2 |
| Toxic effect of tobacco and nicotine | SMC.. | 212899006 | 2 |
| Stop smoking invitation second short message service text message | 9OOB1 | 783441000000103 | 2 |
| Stop smoking invitation short message service text message | 9OOB. | 783381000000101 | 2 |
| Tobacco dependence NOS | E251z | 89765005 | 2 |
| [X]Mental and behavioural disorders due to use of tobacco: other mental and behavioural disorders | Eu17y | 396621000000103 | 2 |
| [X]Mental and behavioural disorders due to use of tobacco: unspecified mental and behavioural disorder | Eu17z | 466861000000102 | 2 |
| Tobacco dependence, episodic | E2512 | 191888003 | 2 |
| Ex smoker | 137S. | 8517006 | 3 |
| Stopped smoking | 137K. | 160617001 | 3 |
| Ex-moderate smoker (10-19/day) | 1379. | 266923002 | 3 |
| Ex-smoker - amount unknown | 137F. | 266928006 | 3 |
| Ex-light smoker (1-9/day) | 1378. | 266922007 | 3 |
| Ex-heavy smoker (20-39/day) | 137A. | 266924008 | 3 |
| Trying to give up smoking | 137G. | 160616005 | 3 |
| Date ceased smoking | 137T. | 160625004 | 3 |
| Ex-cigarette smoker | 137j. | 281018007 | 3 |
| Ex-trivial smoker (<1/day) | 1377. | 266921000 | 3 |
| Ex-very heavy smoker (40+/day) | 137B. | 266925009 | 3 |
| Nicotine replacement therapy | 8B2B. | 313396002 | 3 |
| Referral to stop-smoking clinic | 8HTK. | 315232003 | 3 |
| Smoking cessation therapy | 745H. | 710081004 | 3 |
| Total time smoked | 137n. | 228487000 | 3 |
| Referral to NHS stop smoking service | 8HkQ. | 505281000000106 | 3 |
| Ready to stop smoking | 137b. | 203191000000107 | 3 |
| Cigarette pack-years | 137g. | 401201003 | 3 |
| Ex pipe smoker | 137N. | 160620009 | 3 |
| Smoking cessation milestones | 13p.. | 390900001 | 3 |
| Seen by smoking cessation advisor | 9N2k. | 401068004 | 3 |
| Brief intervention for smoking cessation | 67H6. | 506491000000102 | 3 |
| Referral to smoking cessation service declined | 8IEo. | 871641000000105 | 3 |
| Referral for smoking cessation service offered | 9NS02 | 767641000000109 | 3 |
| Ex cigar smoker | 137O. | 160621008 | 3 |
| Nicotine replacement therapy provided free | 8B3f. | 390905006 | 3 |
| Declined consent for follow-up by smoking cessation team | 9Ndg. | 755741000000100 | 3 |
| Nicotine replacement therapy using nicotine patches | 745H0 | 790121000000107 | 3 |
| Negotiated date for cessation of smoking | 13p0. | 390901002 | 3 |
| Recently stopped smoking | 137K0 | 517211000000106 | 3 |
| Smoking cessation drug therapy | 745H4 | 713700008 | 3 |
| Over the counter nicotine replacement therapy | 8B3Y. | 315055008 | 3 |
| DNA - Did not attend smoking cessation clinic | 9N4M. | 25261000000107 | 3 |
| Ex roll-up cigarette smoker | 137l. | 492191000000103 | 3 |
| Smoking cessation programme start date | 13p5. | 401160008 | 3 |
| Smoking cessation therapy NOS | 745Hz | NA | 3 |
| Referral to smoking cessation service | 8T08. | 871661000000106 | 3 |
| Nicotine replacement therapy refused | 8I39. | 315022003 | 3 |
| Smoking free weeks | 13p4. | 395177003 | 3 |
| Nicotine replacement therapy using nicotine inhalator | 745H2 | 280811000000104 | 3 |
| Ex-smoker annual review - enhanced services administration | 9km.. | 505761000000105 | 3 |
| Other specified smoking cessation therapy | 745Hy | NA | 3 |
| Smoking status at 4 weeks | 13p1. | 390902009 | 3 |
| Stop smoking face to face follow-up | 8HBM. | 505581000000108 | 3 |
| Practice based smoking cessation programme start date | 13p50 | 712971000000108 | 3 |
| Ex-smoker | 137s. | NA | 3 |
| Consent given for follow-up by smoking cessation team | 9Ndf. | 755721000000107 | 3 |
| Nicotine replacement therapy using nicotine gum | 745H1 | 280801000000101 | 3 |
| Carbon monoxide reading at 4 weeks | 13p6. | 413753009 | 3 |
| Smoking status between 4 and 52 weeks | 13p2. | 390903004 | 3 |
| Nicotine replacement therapy using nicotine lozenges | 745H3 | 790131000000109 | 3 |
| Nicotine withdrawal | E023. | 90755006 | 3 |
| Smoking cessation - enhanced services administration | 9kc.. | 374361000000100 | 3 |
| Declined consent for smoking cessation data sharing | 9NdZ. | 751661000000106 | 3 |
| Consent given for smoking cessation data sharing | 9NdW. | 750851000000104 | 3 |
| Declined consent for follow-up evaluation after smoking cessation intervention | 9NdY. | 751101000000101 | 3 |
| Smoking status at 12 weeks | 13p7. | 766931000000106 | 3 |
| Smoking status at 52 weeks | 13p3. | 390904005 | 3 |
| Lost to smoking cessation follow-up | 13p8. | 768971000000109 | 3 |
| Smoking cessation 12 week follow-up | 8HBP. | 850331000000104 | 3 |
| Nicotine replacement therapy provided by community pharmacist | 8BP3. | 200211000000104 | 3 |
| Varenicline therapy | 745H5 | 719591006 | 3 |
| Consent given for follow-up evaluation after smoking cessation intervention | 9NdV. | 750821000000109 | 3 |
| Referred for chronic obstructive pulmonary disease structured smoking assessment - enhanced services administration | 9kf1. | 375851000000108 | 3 |
| Varenicline smoking cessation therapy offered | 8B31G | 966991000000104 | 3 |
| Nicotine replacement therapy contraindicated | 8I2I. | 395174005 | 3 |
| Smoking cessation monitoring template completed - enhanced services administration | 9kc0. | 374391000000106 | 3 |
| [X]Mental and behavioural disorders due to use of tobacco: withdrawal state | Eu173 | 411461000000105 | 3 |
| Issue of nicotine replacement therapy voucher | 8B2B0 | 784481000000108 | 3 |
| Current non-smoker | 137L. | 160618006 | 4 |
| Non-smoker annual review - enhanced services administration | 9kn.. | 505681000000109 | 4 |
| Never smoked tobacco | XE0oh | 266919005 | 5 |
| Non-smoker (& [never smoked tobacco]) | 1371. | 266919005 | 5 |

A1 Supplementary table 5: **Charlson comorbidity index overview**

Full lists can be seen DOI: [10.13140/RG.2.2.14629.42729](http://dx.doi.org/10.13140/RG.2.2.14629.42729). Weights as per: Quan H, Li B, Couris CM, Fushimi K, Graham P, Hider P, et al. Updating and Validating the Charlson Comorbidity Index and Score for Risk Adjustment in Hospital Discharge Abstracts Using Data From 6 Countries. American Journal of Epidemiology. 2011;173(6):676-82. <https://academic.oup.com/aje/article/173/6/676/182985>

| **Collated by Katrina Davis (katrina.davis@kcl.ac.uk) and Ruimin Ma**  **Based on codes in CALIBER (https://www.caliberresearch.org/portal/show/charlson_composite)** | | | | |
| --- | --- | --- | --- | --- |
|  |  | Weights | | |
| Item | Notes | Original | Modified | This study |
| Myocardial infarct | Unchanged from CALIBER | 1 | 0 | 0 |
| Heart failure | Unchanged from CALIBER | 1 | 2 | 2 |
| Peripheral aterial | We noted that the code list did not include procedures relating to abdominal aortic aneurysms, but did not change this | 1 | 0 | 0 |
| Stroke | Unchanged from CALIBER | 1 | 0 | 0 |
| Dementia | Not included | 1 | 2 | 0 |
| Asthma and COPD | Unchanged from CALIBER | 1 | 1 | 1 |
| Connective tissue | Unchanged from CALIBER | 1 | 1 | 1 |
| Peptic ulcer | CALIBER included only gastric disorders, which we widened to include duodenal ulcers and peptic ulcers without position stated | 1 | 0 | 0 |
| Liver | We combined the list for mild and severe illness, as it was felt there was insufficient specificity between the lists | 1 (3) | 2 (4) | 2 |
| Diabetes | Unchanged from CALIBER | 1 | 0 | 0 |
| Paralysis | CALIBER included only hemiplegia, whereas paraplegia is also intended for the charlson, so added | 2 | 2 | 2 |
| Renal failure | Unchanged from CALIBER | 2 | 1 | 1 |
| Complications of diabetes | Unchanged from CALIBER | 2 | 1 | 1 |
| Cancer in last 5 years | We removed the group of codes relating to “history of” cancer given the timing of this item is important | 2 | 2 | 2 |
| HIV | The original item was AIDS, but we have kept the shift in the existing codes to diagnosis of HIV, aware that this is likely to reduce the weighting of this item when we calculate this | 6 | 4 | 4 |
| Cancer with metastases | Unchanged from CALIBER | 6 | 6 | 6 |

A1 Supplementary table 6: **Dementia medication**

| Sub-class | Medication name / Trade name |
| --- | --- |
| Donepezil | Donepezil |
|  | Aricept Evess |
|  | Aricept |
| Memantine | Memantine |
|  | Alzhok |
|  | Ebixa |
|  | Nemdatine |
|  | Marixino |
|  | Valios |
| Other | Galantamine |
|  | Galzemic |
|  | Reminyl |
|  | Acumor XL |
|  | Consion XL |
|  | Elmino XL |
|  | Gaalin |
|  | Galsya XL |
|  | Galzemic XL |
|  | Gatalin XL |
|  | Gazylan XL |
|  | Lotprosin XL |
|  | Luventa XL |
|  | Reminyl XL |
|  | Rivastigmine |
|  | Nimvastid |
|  | Almuriva |
|  | Alzest |
|  | Exelon |
|  | Voleze |
|  | Erastig |

# Appendix 2: [table] Proportion of people with dementia identified by two sources looking over time and by Lambeth residence

Table A2

|  |  |  | **Documentation of dementia diagnosis** | | | | |
| --- | --- | --- | --- | --- | --- | --- | --- |
| Documentation of dementia | total | n. diagnoses per year | Both primary care and specialist | Primary care only | Specialist only | Primary care code (+/- specialist) | In specialist database (+/- primary care) |
| Date of first documentation |  |  | Cohort with Lambeth GP | | | Cohort with Lambeth GP | |
| 2007-2010 | 1578 | *395* | 828 (52%) | 246 (16%) | 504 (32%) | 1074 (68%) | 1332 (73%) |
| 2011-2015 | 2316 | *463* | 1290 (56%) | 489 (21%) | 537 (23%) | 1779 (77%) | 1827 (65%) |
| 2016-2019 | 1345 | *336* | 768 (57%) | 238 (18%) | 339 (25%) | 1006 (75%) | 1107 (70%) |
| **total** | **5239** |  | **2886 (55%)** | **973 (19%)** | **1380 (26%)** | **3859 (74%)** | **4266 (69%)** |
| Date of first documentation |  |  | Cohort with Lambeth GP and address | | | Cohort Lambeth GP and address | |
|  |  |  |  | | |  | |
| 2007-2010 | 1317 | *329* | 713 (54%) | 212 (16%) | 392 (30%) | 925 (70%) | 1105 (72%) |
| 2011-2015 | 1947 | *463* | 1123 (58%) | 414 (21%) | 410 (21%) | 1537 (79%) | 1533 (65%) |
| 2016-2019 | 1126 | *282* | 662 (59%) | 200 (18%) | 264 (23%) | 862 (77%) | 926 (70%) |
| **total** | **4390** |  | **2498 (57%)** | **826 (19%)** | **1066 (24%)** | **3324 (76%)** | **3564 (68%)** |

# Appendix 3: [table] Characteristics and missing data for main cohort from Lambeth DataNet, compared against those also with Lambeth residence.

Table A3 Cohort characteristics – LDN patients with dementia documentation in primary care and/or specialist sources

|  | Main cohort (regardless of residence) n=5239 | Lambeth resident n=4390 |
| --- | --- | --- |
| Age group |  |  |
| 65-74 | 1006 (19%) | 847 (19%) |
| 75-84 | 2379 (45%) | 2012 (46%) |
| 85+ | 1854 (35%) | 1531 (35%) |
| Sex |  |  |
| F | 3131 (60%) | 2593 (59%) |
| M | 2108 (40%) | 1797 (41%) |
| Ethnicity |  |  |
| British | 2057 (39%) | 1683 (38%) |
| Black | 1182 (23%) | 1078 (25%) |
| White | 785 (15%) | 674 (15%) |
| Asian | 281 (5%) | 239 (5%) |
| mixed | 134 (3%) | 120 (3%) |
| other | 56 (1%) | 47 (1%) |
| missing | 744 (14%) | 549 (13%) |
| IMD |  |  |
| Mid-Low deprivation | 3052 (58%) | 2524 (57%) |
| High deprivation | 2076 (40%) | 1866 (43%) |
| missing | 111 (2%) | NA |
| Smoking status |  |  |
| never | 1750 (33%) | 1495 (34%) |
| missing | 651 (12%) | 462 (11%) |
| Comorbidity index |  |  |
| 0 | 1879 (36%) | 1509 (34%) |
| 1 | 1084 (21%) | 901 (21%) |
| 2 to 3 | 1654 (32%) | 1433 (33%) |
| 4 to 5 | 500 (10%) | 439 (10%) |
| 6+ | 122 (2%) | 108 (2%) |
| Consultation rate |  |  |
| Above-average | 2673 (51%) | 2303 (52%) |
| Below average | 1767 (34%) | 1490 (34%) |
| None | 799 (15%) | 597 (14%) |
| Care home |  |  |
| Yes | 380 (7%) | 373 (8%) |
| No | 4859 (93%) | 4017 (92%) |
| Death |  |  |
| During follow-up | 1373 (26%) | 1214 (28%) |
| Not during follow-up | 3866 (74%) | 3176 (72%) |
| Dementia type |  |  |
| Alzheimer's/Mixed | 2863 (55%) | 2359 (54%) |
| Vascular | 1142 (22%) | 984 (22%) |
| Other | 215 (4%) | 186 (4%) |
| missing / unsp | 1019 (19%) | 861 (20%) |
| CRIS record (contact with specialist mental health services) |  |  |
| Yes | 4621 (88%) | 3856 (88%) |
| No | 618 (12%) | 534 (12%) |
| **Total** | **5239** | **4390** |

# Appendix 4: [table] Characteristics of patients with dementia documented in either specialist database or primary care database or both

Table A4 (A-C) Characteristics of patients with dementia documented in either specialist database or primary care database or both. A. Excludes missing data, with proportions indicating the proportion of documented cases (as per Table 1 and 2) B. Accounts for missing data in proportions C. Accounts for missing and restricts to those with prior consultation.

| A. Variable n | | | | |  | B. Full cohort | | | | |  | C. Restricting to patients with prior consultation | | | | |
| --- | --- | --- | --- | --- | --- | --- | --- | --- | --- | --- | --- | --- | --- | --- | --- | --- |
| Proportion with 95% confidence intervals. Variable n due to exclusion of values that are missing. | | | | |  | Proportion and 95% confidence interval. N = 5239 throughout | | | | |  | Proportion and 95% confidence interval. N = 4440 throughout | | | | |
|  |  | Both sources | Specialist only | Primary care only |  |  |  | Both (n.2886) | Specialist (n.1380) | Primary care (n.973) |  |  |  | Both (n.2660) | Specialist (n.900) | Primary care (n.880) |
| Age group (n=5239) | | n.2880 | n.1380 | n.973 |  | Age group (n=5239) | | | | |  | Age group (n=4440) | | |  |  |
|  | 65-74 | 18%  (17-20) | 19%  (17-22) | 22% (19-24) |  |  | 65-74 | 18% (17-20) | 19% (17-22) | 22% (19-24) |  |  | 65-74 | 18% (16-19) | 18% (15-20) | 22% (19-24) |
|  | 75-84 | 47%  (45-49) | 43%  (40-45) | 44%  (41-47) |  |  | 75-84 | 47%  (45-49) | 43%  (40-45) | 44%  (41-47) |  |  | 75-84 | 47%  (46-49) | 41%  (38-44)* | 44%  (40-47) |
|  | 85+ | 34%  (33-36) | 38%  (35-40) | 35%  (32-38) |  |  | 85+ | 34%  (33-36) | 38%  (35-40) | 35%  (32-38) |  |  | 85+ | 35%  (33-37) | 41%  (38-44)^ | 35%  (32-38) |
| Sex (n=5239) | | n.2880 | n.1380 | n.973 |  | Sex (n=5239) | |  |  |  |  | Sex (n=4440) | | |  |  |
|  | Female | 61%  (59-63) | 57%  (55-60) | 59%  (56-62) |  |  | Female | 61%  (59-63) | 57%  (55-60) | 59%  (56-62) |  |  | Female | 61%  (59-63) | 57%  (54-60) | 59%  (56-63) |
|  | Male | 39%  (37-41) | 43%  (40-45) | 41%  (38-44) |  |  | Male | 39%  (37-41) | 43%  (40-45) | 41%  (38-44) |  |  | Male | 39%  (37-41) | 43%  (40-46) | 41%  (37-44) |
| Ethnicity (n=4495) | | n.2662 | n.925 | n.908 |  | Ethnicity (n=5239) | | | | |  | Ethnicity (n=4440) | | |  |  |
|  | British | 44%  (42-46) | 51%  (48-54)^ | 45%  (42-48) |  |  | British | 41%  (39-43) | 34%  (32-37)* | 42%  (39-45) |  |  | British | 41%  (39-43) | 44%  (41-47) | 41%  (38-44) |
|  | Black | 27%  (26-29) | 22%  (19-25)* | 28%  (25-31) |  |  | Black | 25%  (24-27) | 15%  (13-17)* | 26%  (23-29) |  |  | Black | 26%  (25-28) | 18%  (15-20)* | 27%  (25-30) |
|  | White non-British | 18%  (17-20) | 17%  (14-19) | 15%  (13-18) |  |  | White non-British | 17%  (16-18) | 11%  (10-13)* | 14%  (12-17)* |  |  | White non-British | 17%  (16-19) | 14%  (12-16) | 14%  (12-17) |
|  | Asian | 5.7%  (4.9-6.6) | 7.0%  (5.6-8.9) | 7.2%  (5.7-9) |  |  | Asian | 5%  (4-6) | 5%  (4-6) | 7%  (5-8) |  |  | Asian | 5%  (4-6) | 5%  (4-7) | 7%  (5-9) |
|  | Mixed | 3.0%  (2.5-3.8) | 2.8%  (1.9-4.1) | 3%  (2.1-4.3) |  |  | Mixed | 3%  (2-3) | 2%  (1-3) | 3%  (2-4) |  |  | Mixed | 3%  (2-4) | 2%  (2-4) | 3%  (2-4) |
|  | Other | 1.2%  (0.8-1.6) | 0.6%  (0.3-1.4) | 2.1%  (1.3-3.2) |  |  | Other | 1%  (1-2) | 0%  (0-1) | 2%  (1-3) |  |  | Other | 1%  (1-2) | 0%  (0-1) | 2%  (1-3) |
|  | Missing | omitted | omitted | omitted |  |  | Missing | 8%  (7-9) | 33%  (31-35)^ | 7%  (5-8) |  |  | Missing | 6%  (5-7) | 17%  (14-19)^ | 6%  (5-8) |
| Deprivation (IMD) (n=5128) | | n.2829 | n.1345 | n.954 |  | Deprivation (IMD) (n=5239) | |  |  |  |  | Deprivation (IMD) (n=4440) | |  |  |  |
|  | Quintile 2-5 | 59%  (57-61) | 60%  (57-63) | 60%  (57-63) |  |  | Quintile 2-5 | 58%  (56-60) | 58%  (56-61) | 59%  (56-62) |  |  | Quintile 2-5 | 59%  (57-61) | 59%  (56-62) | 59%  (56-62) |
|  | Quintile 1 (most deprived) | 41%  (39-43) | 40%  (37-43) | 40%  (37-43) |  |  | Quintile 1  (most deprived) | 40%  (38-42) | 39%  (37-42) | 39%  (36-42) |  |  | Quintile 1  (most deprived) | 39%  (37-41) | 39%  (36-42) | 39%  (36-42) |
|  | Missing address | omitted | omitted | omitted |  |  | Missing address | 2%  (2-3) | 3%  (2-4) | 2%  (1-3) |  |  | Missing address | 2%  (2-3) | 2%  (1-3) | 2%  (1-3) |
| Smoking (n=4589) | | n.2770 | n.878 | n.941 |  | Smoking (n=5239) | |  |  |  |  | Smoking (n=4440) | |  |  |  |
|  | Never | 37%  (35-38) | 43%  (40-46)^ | 37%  (34-40) |  |  | Never | 35%  (33-37) | 27%  (25-30)* | 35%  (32-38) |  |  | Never | 34%  (33-36) | 34%  (31-37) | 34%  (31-37) |
|  | Former / mixed | 48%  (46-50) | 37%  (34-40)* | 46%  (43-49) |  |  | Former / mixed | 46%  (44-48) | 24%  (21-26)* | 44%  (41-47) |  |  | Former / mixed | 47%  (45-49) | 33%  (30-36)* | 45%  (42-48) |
|  | Current | 15%  (14-17) | 20%  (17-22) | 18%  (15-20) |  |  | Current | 15%  (14-16) | 13%  (11-14) | 17%  (15-20) |  |  | Current | 16%  (14-17) | 17%  (15-20) | 18%  (16-21) |
|  | Missing | omitted | omitted | omitted |  |  | Missing | 4%  (3-5) | 36%  (34-39)^ | 3%  (2-5) |  |  | Missing | 3%  (3-4) | 16%  (14-19)^ | 3%  (2-4) |
| Primary care consultations (n=5239) (a) | | | | |  | Primary care consultations (n=5239) (a) | | | | |  | Primary care consultations (n=4440) (a) | | | | |
|  | Above average | 59%  (57-60) | 36%  (34-39)* | 50%  (47-53)* |  |  | Above average | 59%  (57-60) | 36%  (34-39)* | 50%  (47-53)* |  |  | Above average | 63%  (62-65) | 56%  (52-59)* | 55%  (52-58)* |
|  | Average or less | 34%  (32-35) | 29%  (27-31)* | 41%  (38-44)^ |  |  | Average or less | 34%  (32-35) | 29%  (27-31)* | 41%  (38-44)^ |  |  | Average or less | 37%  (35-38) | 44%  (41-48)^ | 45%  (42-48)^ |
|  | None | 8%  (7-9) | 35%  (32-37)^ | 10%  (8-12) |  |  | None | 8%  (7-9) | 35%  (32-37)^ | 10%  (8-12) |  |  | None | omitted | | |
| Care home residence (n=5239) (a) | | | | |  | Care home residence (n=5239) (a) | | | | |  | Care home residence (n=4440) (a) | | | | |
|  | Yes | 8%  (7-9) | 3%  (3-4)* | 11%  (9-13) |  |  | Yes | 8%  (7-9) | 3%  (3-4)* | 11%  (9-13) |  |  | Yes | 7%  (6-8) | 4%  (3-6) | 11%  (9-13)^ |
|  | No | 92%  (91-93) | 97%  (96-97)^ | 89%  (87-91) |  |  | No | 92%  (91-93) | 97%  (96-97)^ | 89%  (87-91) |  |  | No | 93%  (92-94) | 96%  (94-97) | 89%  (87-91)* |
| Comorbidity index (n=5239)(a)(c) | | | | |  | Charlson comorbidity index(n=5239)(a)(c) | | | | |  | Charlson comorbidity index(n=4440)(a)(c) | | | | |
|  | 0 | 31%  (30-33) | 47%  (45-50)^ | 33%  (30-36) |  |  | 0 | 31%  (30-33) | 47%  (45-50)^ | 33%  (30-36) |  |  | 0 | 28%  (27-30) | 34%  (31-37)^ | 30%  (28-34) |
|  | 1 | 22%  (20-23) | 20%  (18-22) | 19%  (17-22) |  |  | 1 | 22%  (20-23) | 20%  (18-22) | 19%  (17-22) |  |  | 1 | 22%  (20-23) | 23%  (21-26) | 19%  (17-22) |
|  | 2 to 3 | 34%  (33-36) | 24%  (22-27)* | 34%  (31-37) |  |  | 2 to 3 | 34%  (33-36) | 24%  (22-27)* | 34%  (31-37) |  |  | 2 to 3 | 36%  (34-38) | 31%  (28-34) | 35%  (32-38) |
|  | 4 to 5 | 10%  (9-12) | 7%  (6-8)* | 11%  (9-13) |  |  | 4 to 5 | 10%  (9-12) | 7%  (6-8)* | 11%  (9-13) |  |  | 4 to 5 | 11%  (10-13) | 10%  (8-12) | 12%  (10-14) |
|  | 6+ | 2.3%  (1.8-2.9) | 1.7%  (1.2-2.6) | 3.4%  (2.4-4.7) |  |  | 6+ | 2.3%  (1.8-2.9) | 1.7%  (1.2-2.6) | 3.4%  (2.4-4.7) |  |  | 6+ | 2%  (2-3) | 2%  (2-4) | 4%  (3-5) |
| Mortality(n=5239)(b) | | | | |  | Mortality(n=5239)(b) | | | | |  | Mortality(n=4440)(b) | | | | |
|  | No | 76%  (75-78) | 72%  (70-75) | 69%  (66-72)* |  |  | Alive | 76%  (75-78) | 72%  (70-75) | 69%  (66-72)* |  |  | Alive | 76%  (75-78) | 63%  (60-66)* | 68%  (65-71)* |
|  | Yes | 24%  (22-25) | 28%  (25-30) | 31%  (28-34)^ |  |  | Death within 4y | 24%  (22-25) | 28%  (25-30) | 31%  (28-34)^ |  |  | Death within 4y | 24%  (22-25) | 37%  (34-40)^ | 32%  (29-35)^ |
| Subtype (n=4220) | | n.2644 | n.1026 | n.550 |  | Subtype (n=5239) | |  |  |  |  | Subtype (n=4220) | |  |  |  |
|  | Alzheimers / mixed | 74%  (73-76) | 60%  (57-63)* | 52%  (48-56)* |  |  | Alzheimers / mixed | 68%  (66-70) | 44%  (42-47)* | 29%  (27-32)* |  |  | Alzheimers / mixed | 69%  (67-71) | 38%  (34-41)* | 29%  (26-32)* |
|  | Vascular | 21%  (20-23) | 34%  (31-37)^ | 41%  (37-45)^ |  |  | Vascular | 20%  (18-21) | 25%  (23-28)^ | 23%  (21-26)^ |  |  | Vascular | 19%  (18-20) | 27%  (24-30)^ | 21%  (19-24) |
|  | Other specified | 4.3%  (3.6-5.2) | 6%  (4.7-7.7) | 7.1%  (5.2-9.5) |  |  | Other specified | 4%  (3-5) | 4%  (4-6) | 4%  (3-5) |  |  | Other specified | 4%  (3-5) | 5%  (4-7) | 4%  (3-5) |
|  | Unspecified | omitted | omitted | omitted |  |  | Unspecified | 8%  (7-9) | 26%  (23-28)^ | 43%  (40-47)^ |  |  | Unspecified | 8%  (7-9) | 30%  (28-34)^ | 46%  (43-49)^ |
| Dementia medication(n=5239)(b) | | | | |  | Dementia medication(n=5239)(b) | | | | |  | Dementia medication(n=4440)(b) | | | | |
|  | No | 59%  (57-61) | 92%  (91-94)^ | 77%  (74-80)^ |  |  | No | 59%  (57-61) | 92%  (91-94)^ | 77%  (74-80)^ |  |  | No | 58%  (56-60) | 89%  (87-91)^ | 77%  (74-79)^ |
|  | Yes | 41%  (39-43) | 8%  (6-9)* | 23%  (20-26)* |  |  | Yes | 41%  (39-43) | 8%  (6-9)* | 23%  (20-26)* |  |  | Yes | 42%  (40-44) | 11%  (9-13)* | 23%  (21-26)* |

a) As documented before the first dementia documentation

b) In the four years post-first dementia documentation, or august 2019 if earlier

c) Modified Charlson comorbidity index from primary care-coded morbidities and weights in Quan et al.

^ 95% confidence intervals suggest above proportion for those in both databases (not formally tested, no correction for multiple comparisons)

* 95% confidence intervals suggest below proportion for those in both databases (not formally tested, no correction for multiple comparisons)

# Appendix 5: [table&figure] Prescription of dementia medication in primary care by source of dementia documentation and subtype of dementia

Table A5: Proportion of patients prescribed dementia medication grouped by documentation source and subtype of dementia (bars are 95% confidence intervals)

|  | n. prescribed dementia medications / N. patients in category,  proportion prescribed dementia medication (95% confidence intervals) | | | |  |  |
| --- | --- | --- | --- | --- | --- | --- |
|  | source of dementia identification | | | | |  |
| Subtype | both specialist and primary care | specialist database only | primary care codes only | **Overall** | |  |
| Alzheimers / Mixed | 1022 / 1963 | 64 / 614 | 143 / 286 | **1229 / 2863** | |  |
|  | 52% (50-54) | 10% (8-13) | 50% (44-56) | **43% (41-45)** | |  |
| Vascular | 53 / 567 | 12 / 350 | 12 / 350 | **83 / 1142** | |  |
|  | 9% (7-12) | 3% (2-6) | 8% (5-12) | **7% (6-9)** | |  |
| Other specified | 53 / 567 | 12 / 62 | 18 / 39 | **88 / 215** | |  |
|  | 51% (42-60) | 19% (11-31) | 46% (32-61) | **41% (35-48)** | |  |
| Unspecified or missing | 58 / 114 | 19 / 354 | 43 / 423 | **105 / 1019** | |  |
|  | 18% (13-23) | 5% (3-8) | 10% (8-13) | **10% (9-12)** | |  |
| **Overall** | **1176 / 2886** | **107 / 1380** | **222 / 973** | **1505 / 5239** | |  |
|  | **41% (39-43)** | **8% (6-9)** | **23% (20-26)** | **29% (28-30)** | |  |

Figure A5: Proportion of patients prescribed dementia medication grouped by documentation source and subtype of dementia (bars are 95% confidence intervals)
